# An objective comparison of two pulse oximetry sensors with different adhesive systems on healthy human volunteers based on biophysical assessments

**DOI:** 10.1111/srt.13212

**Published:** 2022-11-03

**Authors:** Gary Grove, Timothy Houser, Jacob Dove, Derek Moody

**Affiliations:** ^1^ cyberDERM, Inc. Broomall Pennsylvania USA; ^2^ Dermico LLC Broomall Pennsylvania USA; ^3^ Research and Development Patient Monitoring Medtronic Boulder Colorado USA

**Keywords:** gentleness, medical adhesive related skin injuries, sensor reapplication, silicone adhesive, trans‐epidermal water loss

## Abstract

**Background:**

Medical Adhesive Related Skin Injuries can arise from topically applied medical devices, especially in those with fragile skin, including the elderly and premature infants. The purpose of this study was to compare gentleness and reapplication of two pulse oximetry sensors (OxySoftN and MaxN, Medtronic, Boulder, CO).

**Materials and methods:**

Eighteen healthy subjects aged 65 years and older were enrolled in the gentleness trial, and 20 healthy subjects (18–69 years) were enrolled in the reapplication trial. For the gentleness trial, trans‐epidermal water loss (TEWL) measurements were made at five sites on each forearm at three time points (baseline [T0], 4‐h postinitial wear [T1], 4‐h postsecond wear [T2]). Total amount of protein adhered to each device was also determined. For the reapplication trial, a series of 180° peel tests were performed to observe the forces required to detach the sensor from the skin.

**Results:**

TEWL rates in the tail region were significantly greater with MaxN compared to OxySoftN at T1 (*p* < 0.05). Both were significantly greater than control (*p* < 0.05). Further, protein analysis revealed that the amount of protein removed was significantly less with OxySoftN compared to MaxN (*p* < < 0.0001). Differences in loss of adhesion of the tail region between the two sensors were demonstrated, with OxySoftN depreciating at a much slower rate compared with MaxN.

**Conclusion:**

The OxySoftN sensor appears to be gentle, even on fragile skin, based on reduced strain on the skin during removal. Further, it demonstrated the ability to withstand several reapplications without functional loss in adhesion.

## 1 INTRODUCTION

It is generally recognized that Medical Adhesive Related Skin Injuries (MARSI) are very common and can occur with any medical device that adheres to the skin. Elderly and premature infants are at particular risk for skin injuries based on their fragile skin.^1^ Premature infants are at greatest risk as their skin is not fully developed.^2^

Pulse oximetry is a standard of care that is routinely used in the NICU on premature infants.^3^ The highest performing pulse oximetry systems utilize a sensor that adheres to the skin with an adhesive. The adhesive creates good tissue‐sensor optical coupling and maintains that coupling during challenging conditions, such as motion.^4^ During use, pulse oximetry sensors require frequent skin checks that involve moving the sensor on fragile skin, such as the skin on neonates. These skin checks can be as frequent as every 3 h for the most at risk patients. Hence, it is desirable to have pulse oximetry sensors that can be repositionable and gentle on removal.

Measurement of gentleness of an adhesive is based on measuring the disruption of the skin barrier. A consensus group of key opinion leaders recommends both trans‐epidermal water loss (TEWL) and the amount of protein removed as highly objective measures to clinically assess and document MARSI.^1^ This is especially true for TEWL as it has been well documented that the removal of the outermost layers of the stratum corneum disrupts the skin barrier function, which leads to elevated TEWL rates.^5^, ^6^, ^7^, ^8^, ^9^ Thus, measuring TEWL rates before and after adhesive removal can show the effect on skin barrier function due to the removal of the adhesive. The amount of skin that is removed when an adhesive is removed relates both to how gentle the removal is and the amount of skin disruption.^10^, ^11^, ^12^ The amount of skin that is removed can also be measured by washing the removed adhesive and then measuring the amount of protein that is present. Any protein found on the previously removed adhesive is assumed to come from the skin to which it was adhered.

The purpose of the current study was to compare the gentleness and reapplication of two pulse oximetry sensors, OxySoftN and MaxN (Medtronic, Boulder, CO). OxySoftN uses a new silicone‐based skin‐contacting adhesive, whereas MaxN uses an acrylic adhesive in the optical region and synthetic rubber adhesive in the tail region. The ability to reapply the sensor is characterized through peel force measurements on skin. The gentleness is characterized through TEWL and protein removal. Significant benefits were observed with the silicone‐based patient‐side adhesive.

## 2 MATERIALS AND METHODS

Two studies comparing different attributes of OxySoftN and MaxN in a pair‐wise fashion were carried out on healthy volunteers and performed in accordance with the Declaration of Helsinki under protocols that were duly approved by an independent review board (Integreview IRB, Austin, TX).

All volunteers were fully informed about the purpose of the study in which they were participating and the associated instrumental methods. Upon signing an informed consent document, they were instructed to stop the use of all potentially moisturizing products (lotions, sunscreens, insect repellants, etc.) on their arms for the 3 days prior to the study day. Upon arrival at the clinic, any hair within the test sites, which could interfere with the procedures, was removed using a gentle surgical prep clipper. Both volar forearms were then wiped with an alcohol swab and allowed to dry for at least 2 min before mapping out the test sites using a nontoxic marker.

### 2.1 Participants

Eighteen (18) healthy subjects (five males and 13 females) were enrolled in the gentleness study. All were aged 65 years or older, with the assumption being that they might be more likely to have more fragile skin than those below age 65 years.^1^ Twenty (20) healthy subjects (three males and 17 females) between 18 and 69 years of age were enrolled in the reapplication study.

### 2.2 Investigational products

**FIGURE 1:**
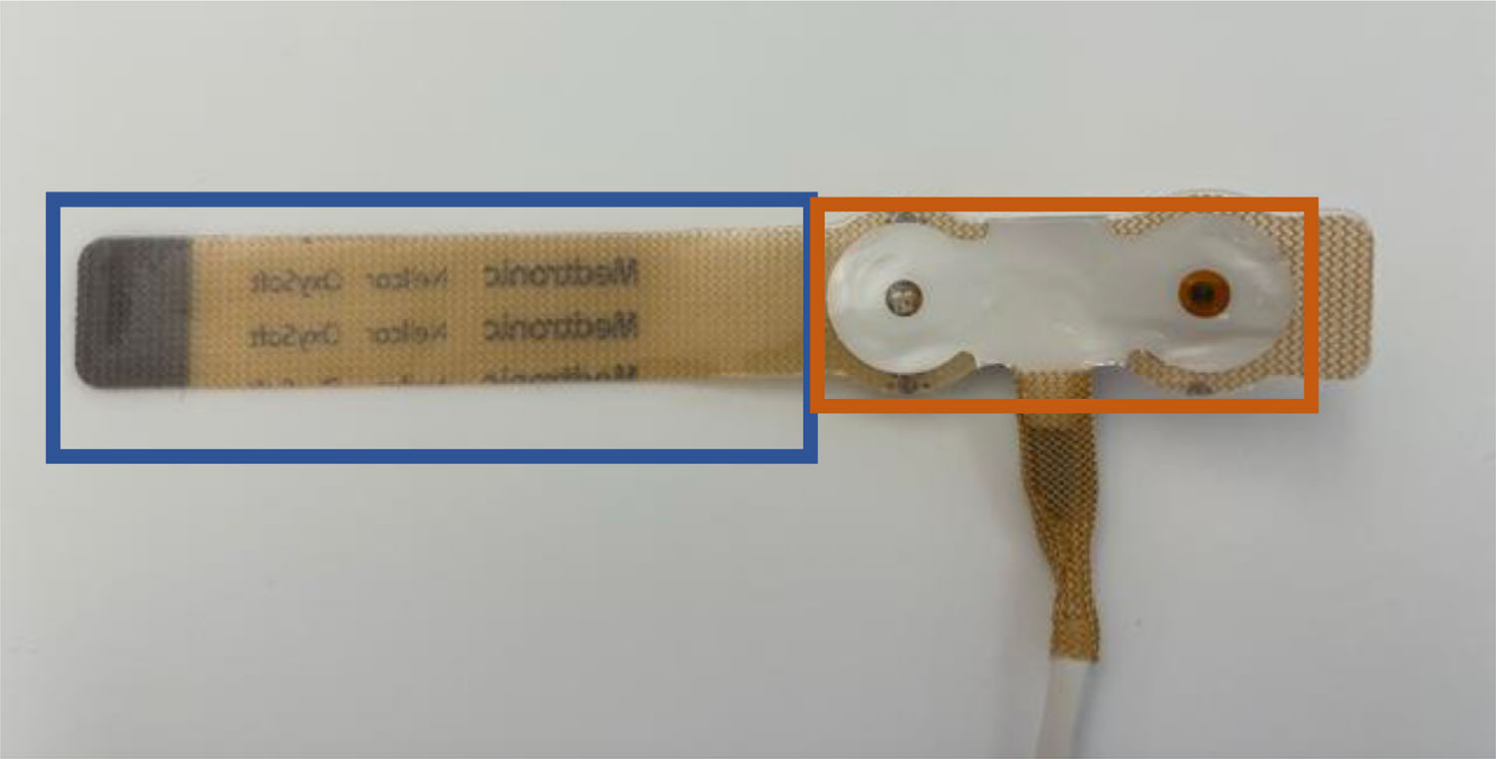
Image of optical (outlined in orange) and tail (outlined in blue) regions on the OxySoftN sensor. OxySoftN uses a silicone‐based skin‐contacting adhesive.

The test products for both studies were provided by Medtronic (Boulder, CO). Both devices are pulse oximetry sensors that are very similar in design, consisting of an optical region that houses the sensor and an associated fabric tail, as shown in Figure 1. The primary difference between the two sensors is that the MaxN sensor uses a synthetic rubber and acrylic patient‐contacting adhesives for the skin interface, while the OxySoftN uses a silicone adhesive system. Each device was identified by subject number and test site location for tracking purposes.

### 2.3 Procedures for gentleness study

This was a 1‐day study in which subjects remained at the clinic for approximately 10 h.

TEWL measurements were made using a recently calibrated cyberDERM RG1 Evaporimeter System (Broomall, PA) with dual TEWL Probes that were manufactured by Cortex Technology (Hadsund, Denmark) and available in the US through cyberDERM, Inc. (Media, PA). All the measurements were performed in an environmentally controlled room with temperature at 20–22°C and relative humidity at 40%–60%. Measurements were taken after a 30‐min acclimatization period, with the test areas on the volar forearm fully exposed. The TEWL rate was calculated as the average value over a 20 s period after steady‐state conditions had been reached.

**FIGURE 2:**
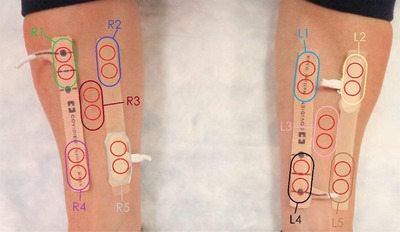
Physical location of the 10 labeled measurement sites with their specific designations. The red circles represent footprint of the paired trans‐epidermal water loss (TEWL) probes within each measurement area.

Measurements were made on the five (5) sites that have been mapped out on each arm as labeled in Figure 2. This is a kitty‐corner design in which the test devices are attached to the skin diagonally opposed in a paired fashion. In this case, R1 and L4 represent the optical region, and L1 and R4 represent the tail region of one of the test devices. For the other test device R5 and L2 represent its optical region while R2 and L5 represents its tail region. A common central area between the two devices (R3 or L3) serves as the control for that arm. The red circles outline the measurement area covered by each of the paired TEWL probes. The assignments of the test devices to the medial or lateral sides of the forearms were done according to a randomization map that maintains this balanced design.

TEWL measurements were taken at the following time points:

T0: Baseline (prior to the first application)
T1: After 4 h of wear (1st application of devices)
T2: After 4 h of wear (2nd application of devices)

At T1, after the devices had been worn for approximately 4 h, the devices were manually removed and retained for reapplication for the second wear period. After the 30‐min post‐removal TEWL measurements were completed, the same devices were reapplied to the same sites as at the baseline visit. Approximately 25% of the MaxN sensors showed some signs of lifting while this never was a problem with the OxySoftN sensors. In a few cases, there were concerns that the MaxN device may become detached completely, and these sensors were replaced during the second wear period. At T2, the devices were again manually removed and then sent to Nelson Labs (Salt Lake City, UT), where the total amount of protein adhered to each device was determined using the Micro BCA^tm^ Protein Test.

### 2.4 Procedures for reapplication study

This was a 1‐day study in which the subjects remained at the clinic for approximately 2 h.

**FIGURE 3:**
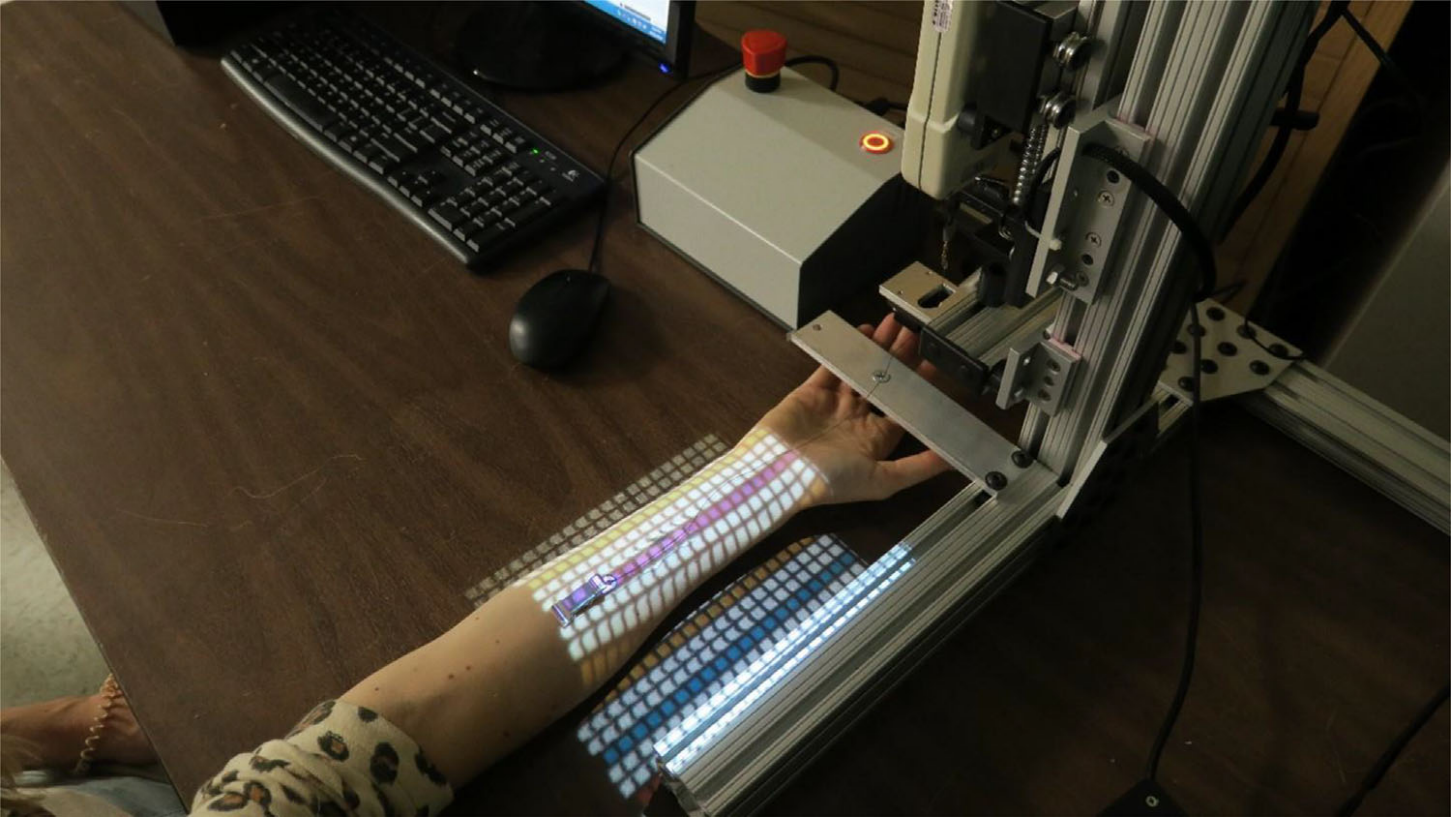
The computerized cyberDERM 180° peel tester uses a stepper motor attached to uniaxial lead screw to pull the stressing clamp at a constant rate of 5.0 + 0.2 mm/s. A calibrated Imada Digital Force Gauge records the resulting tension at least one sample per mm tape peeled.

The computerized cyberDERM 180° peel tester (Figure 3) is designed according to PSTC 101 section 5.5 Adhesion Tester specifications 5 and uses a stepper motor attached to a uniaxial lead screw to pull the stressing clamp at a constant rate of 5.0 + 0.2 mm/s. A calibrated Imada Digital Force Gauge records the resulting tension at least one (1) sample per mm tape peeled. The scale range is such that the expected mean test level falls between 20% and 80% of the full scale. A series of tests using physical standards and appropriate exemplars of medical adhesives being peeled from stainless steel have been run to assure that the current configuration meets these specifications and is suitable for these in vivo studies.

**FIGURE 4:**
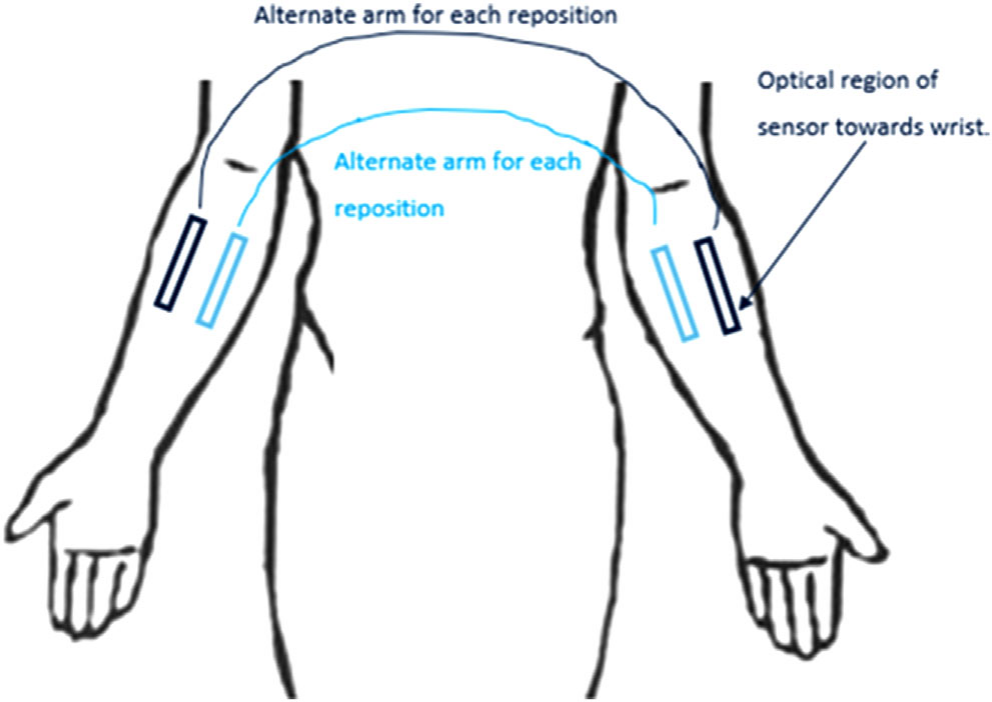
Devices were tested in a pairwise fashion, with random assignment to lateral or medial location of the forearm. The optical sensor region was always positioned toward the wrist. Peel tests alternated between right and left forearms, with the first peel test always starting on the lateral site of the right forearm, until 18 peels tests were completed.

The OxySoftN and MaxN devices were run in a pairwise fashion, with one being assigned to the lateral side and the other to the medial side of the forearm according to the randomized treatment map, with the optical sensor region always positioned toward the wrist as shown in Figure 4. The first peel test was always started on the lateral site of the right and then moved to the left lateral forearm for the second peel test and then alternating to the same location on the opposite arm until 18 peel tests have been completed with that device. This alternating sequence was then run with the other device starting at the medial site on the right and then the left medial forearm and so on until another series of 18 peels tests have been completed with that device as well.

A nontoxic marker was used to place fiducial marks on the subject's skin to ensure the device was placed parallel to the major axis of the forearm. The subject's arm was then positioned within the Peel Test Apparatus such that the test site surface was in plane with the horizontal path that the clamp would take during the run. A micro projector attached to the apparatus overlaid an image of the peel traverse to ensure proper alignment. Extreme care was also taken to have the subject always have his fingers properly placed and arm fully extended so that the tension across the skin surface remained reasonably constant during the entire series of peels for that individual.

**FIGURE 5:**
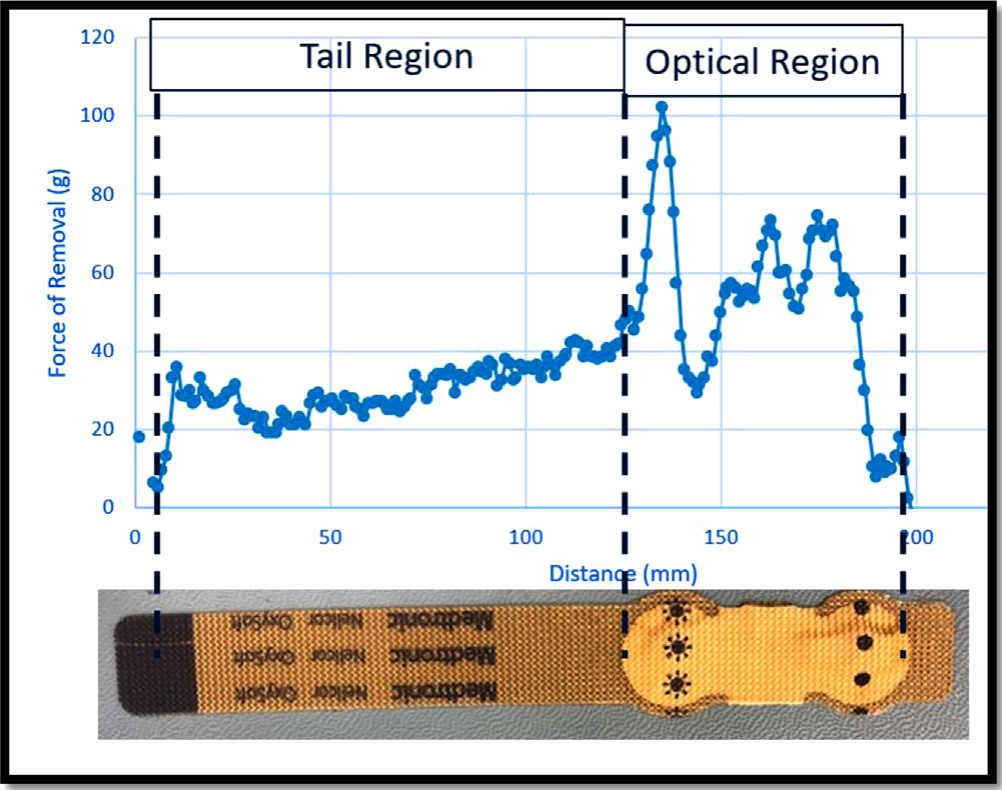
An example plot of the differences in the amount of force required to remove the tail and optical regions of the device during the 180° peel test, due to differences in stiffness along the device

One caveat with the 180° peel test is that observed forces arise not only from detaching the adhesive device from the skin but also from the device bending back upon itself. Since both test devices consist of a flexible lead strip and a stiffer section with electrodes, the observed plot, as shown in Figure 5, is much more complex than typically seen with a uniformly coated homogenous adhesive test strip. For the tail region, the mean peel adhesion value was calculated using at least 50 data points from the steady‐state region for that sample run.

As the adhesive under the optical region is the same as the adhesive on the tail for both devices, the increase in values seen in the optical region is not an increase in adhesion but rather an increase in force of removal due to the work required to bend the stiff components. Nevertheless, we do want to analyze this portion of the peel test because the effectiveness of the device depends upon the optical sensors being firmly attached to the skin. Thus, in addition to confirming visually that the sensor is still firmly attached to the skin, we also determined the work required to remove the optical region by using the Riemann left sum as an approximation of the area under the curve within the optical region.

### 2.5 Statistical analysis

The data were processed using GraphPad statistical software. A two‐sided *p*‐value of <0.05 was considered statistically significant in all tests. Continuous variables are presented as mean ± standard deviation, unless otherwise noted.

For the TEWL results, the appropriate values were pooled by individual subject to provide a mean value for the optical region and tail regions of the two sensors and the corresponding control for each time point. A repeated measure ANOVA with Tukey‐Kramer Multiple Comparisons Test was then run on the net change from baseline to determine the degree of significance at T1 and T2.

For protein adhered to each device, a paired *t*‐test was employed. A value of 1.1 gm was given to those samples falling below the detection limits of the Micro BCA^tm^ assay.

For the Peel Test, the appropriate values for the optical and tail regions were calculated from profiles obtained from both devices for each of the 18 serial cycles of removal and reattachment from each subject. These were then compiled, and the mean values plotted as a function of cycle number. The resulting trendlines, which show the dynamics of decreasing adhesion with increased number of reapplications, were analyzed for goodness of fit using the MS Excel toolbox.

## 3 RESULTS

### 3.1 Gentleness

**FIGURE 6:**
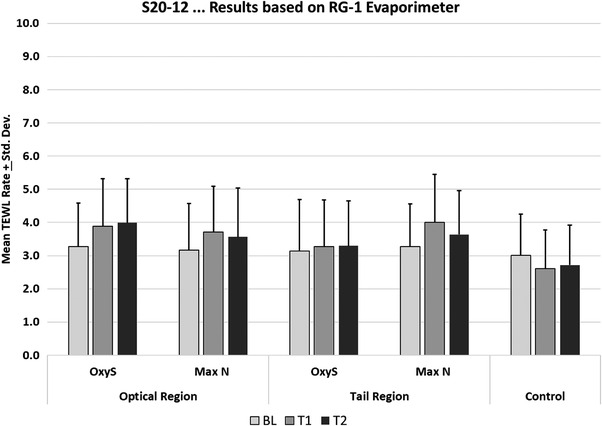
Mean trans‐epidermal water loss (TEWL) rates for the respective optical and tail regions of the OxySoftN and MaxN sensors at baseline and after the first 4‐h wear period (T1) or the second 4‐h wear period (T2)

Mean TEWL rates are summarized in Figure 6. For both T1 and T2, TEWL rates were significantly elevated but to similar extents in those sites from which the optical region of either the OxySoftN or MaxN sensors had been removed. For the tail region, TEWL rates were only significantly elevated in those sites from which the MaxN sensor was removed.

In comparing results from the optical region of the OxySoftN and MaxN sensors, it was found that at both T1 and T2, TEWL rates were significantly elevated with both devices compared to the control sites. However, no significant differences were found to exist between the two sensors at either time point.

In comparing the results from the tail region of the OxySoftN and MaxN sensors, it was found that at T1, TEWL rates were significantly elevated for both the OxySoftN and MaxN sensors when compared to the control (*p* < 0.05). The TEWL rates were also significantly greater with the MaxN sensor compared to OxySoftN at T1 (*p* < 0.05).

In comparing the results obtained at T2 from the tail region, it was found that TEWL rates were significantly elevated only for the MaxN sensors when compared to the control (*p* < 0.01). TEWL rates were not significantly increased for the OxySoftN sensors when compared to control (*p* > 0.05).

**FIGURE 7:**
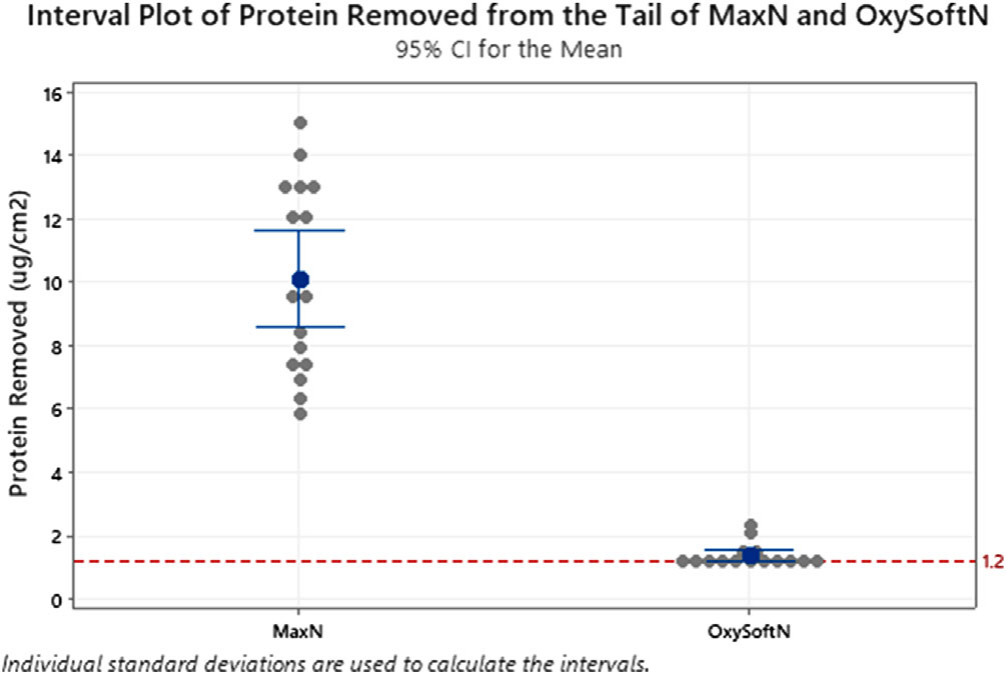
Interval plot comparing amount of protein adhered to the removed device. Samples for subject #16 were not suitable for measurement. The detection limit of the test was 1.2 μg/cm^2^.

Further, the amount of protein removed was significantly less with the OxySoftN sensor compared to the MaxN sensor (1.3 ± 0.4 ug/ml vs. 10.1 ± 2.9 μg/ml, *p* < < 0.0001), as shown in Figure 7. Indeed, nearly 40% of the OxySoftN samples fell below the minimum detection limit of the analytical method used by Nelson Laboratories, which means that the true differences in protein removed between the two sensors are greatly underestimated.

### 3.2 Reapplication

**FIGURE 8:**
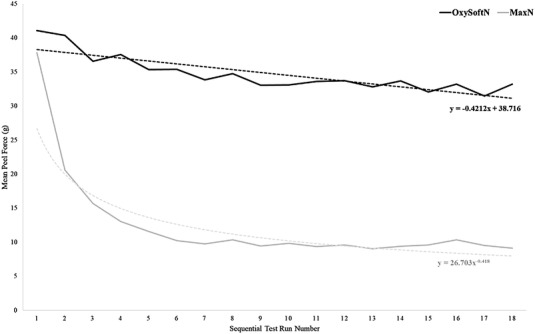
Mean peel force (g), as measured at the tail region, required to remove OxySoftN and MaxN sensors during the 180° peel test at each test run. The black solid line represents the mean peel force for each test run of the OxySoftN sensor. The dotted black line represents the decay trendline for the OxySoftN sensor. The gray solid line represents the mean peel force for each test run of the MaxN sensor. The dotted gray line represents the decay trendline for the MaxN sensor.

**FIGURE 9:**
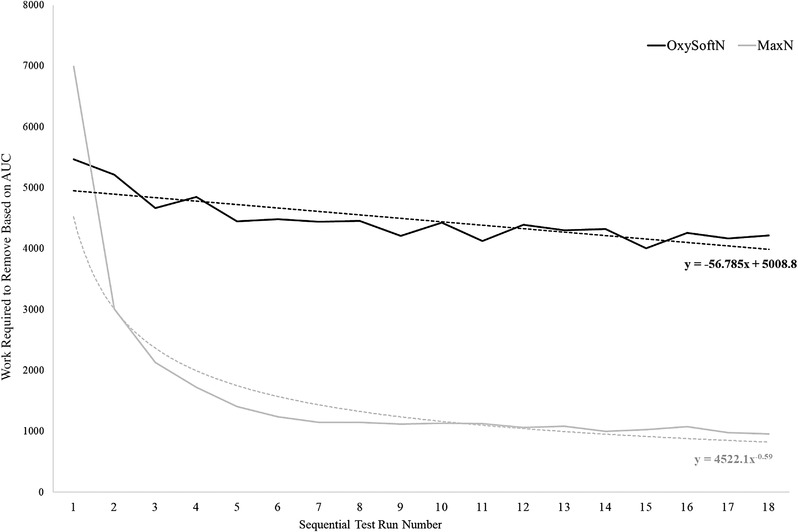
Work required, as measured at the optical region, to remove OxySoftN and MaxN sensors during the 180° peel test at each test run based on area under the curve. The black solid line represents mean work for each test run of the OxySoftN sensor. The dotted black line represents the decay trendline for the OxySoftN sensor. The gray solid line represents mean work for each test run of the MaxN sensor. The dotted gray line represents the decay trendline for the MaxN sensor.

Successful completion of all 18 reapplication trials was seen with the OxySoftN sensor. However, in 60% of cases, the MaxN sensor lost adhesion prior to the 18th trial. To gain a better insight into this observation, we compiled the calculated values for the 18 sequential runs sorted by code for the 20 individuals who participated in this study. The group averages for each device obtained from the tail and optical regions were then plotted as a function of the sequential run number (Figures 8 and 9). The resulting trendlines demonstrated decreased adhesion with serial cycles of removal and reattachment is dramatically different for the two sensors. The loss in adhesion of the tail region of OxySoftN decreases linearly at a much slower and constant rate compared with the MaxN, whose adhesion drops rather precipitously early on and rapidly approaches an asymptote after five or six cycles (Figure 8). This observation is also apparent when the composite graph is based on the work required to remove the optical region, as shown in Figure 9.

It should be appreciated that interpreting the data obtained from the optical region is complicated, as factors other than the adhesion can impact the work required to remove the device from the skin. Chief among these is that this region contains the sensors, causing it to be stiffer, and thus more work is required to bend the region back on itself to achieve the 180° pull. Nevertheless, we feel that it is the loss of adhesion that is primarily responsible for the observed behavior.

## 4 DISCUSSION

The silicone‐based adhesive of the OxySoftN sensor does not appear to disrupt the skin barrier function, as evidenced by the TEWL results. Although TEWL rates were significantly elevated in those sites to which either the OxySoftN or MaxN sensors were attached compared to the intact skin of the control sites, it is important to understand that the degree of change, although statistically significant, is clinically very modest and iin‐line with previous findings.^9^ The new soft silicone adhesive is significantly different and gentler when compared to the acrylic of the MaxN, which is the adhesive in the optical region. There was no difference in TEWL between the two sensors seen in the tail region that has synthetic rubber on MaxN.

The protein removal results also demonstrated that the OxySoftN sensor removes much less skin than the MaxN. The amount of protein removal was significantly different, and the real difference is probably even larger, as the OxySoftN fell below the detection limit of the test. Lower dilution ratios than those used here would increase the concentration of protein in the assay, potentially allowing for a lower detection limit of protein on future adhesives. The results from the protein assay demonstrate that the OxySoftN adhesive removed less skin than the MaxN sensor, corroborating the difference in TEWL seen between the two devices in the sensor region.

Peel force testing demonstrated that the adhesive on the OxySoftN sensor far outlasts the MaxN sensor. The OxySoftN adhesive was able to reposition up to 18 times, whereas the MaxN lost adhesion more quickly. The decay rate of adhesion can be estimated through the fitted parameters. The adhesion of OxySoftN decayed by 1.1% per reposition for both the tail and optical region. The typical lifetime of an adhesive is 1–3 days. Assuming the sensor is removed and placed at a new site every 4 h, 18 reapplications would not be uncommon. Here, OxySoftN retained adhesion over 18 removals and, on average, decreased by approximately 20%. This would also suggest that limit for re‐adhering the OxySoftN sensor was not achieved, even at 18 repositions.

The reapplication testing was completed in rapid succession and therefore did not have a 4‐h dwell time, as would be expected in the NICU. However, increasing the dwell time would likely not impact the results for OxySoftN but increase the decay rate for the MaxN. Traditional adhesives, such as the MaxN, exhibit increased peel forces and reduced reapplication with longer dwell times as the adhesive can flow and fully wet the skin.^13^ The increased adhesion increases the likelihood of removing skin, which decreases the ability to re‐adhere due to a decreased surface area of clean adhesive. Silicone adhesives do not build adhesion over time as this type of adhesive can fully wet the skin on the initial application.^14^ Hence, silicone adhesives have similar peel force values for short or long dwell times.

Skin‐contacting adhesives form physical bonds with the skin, which requires the adhesive to be in close contact with the skin.^13^ Particulates that adhere to the adhesive inhibit the ability of the adhesive to then adhere to the skin. The fact that OxySoftN removes less skin, as demonstrated through protein analysis, would be a benefit for retaining adhesion to skin over multiple reapplications. Less skin cells adhered to the OxySoftN sensor suggesting that there is more adhesive area under the sensor that can adhere to the skin. The MaxN removed more skin and therefore skin covering a large amount of its surface area. This reduces the amount of surface area that can adhere to the subject and therefore reduces the peel force after being repositioned. Waring et al. show this effect on a silicone adhesive, with higher protein assay results correlated to higher contamination on the adhesive.^10^ These gentleness and reapplication results are consistent with expectations of a silicone versus traditional acrylic and synthetic rubber adhesives. Silicone adhesives can be strong adhesives that still allow for reduction in skin injuries. Silicone gel adhesives allow for gentleness based on their physical properties that reduce the strain on the skin during removal.^1^ This allows silicone adhesives to be both strong and gentle. Gentle adhesives also allow for more reapplications, as they do not degrade or become covered in skin upon removal.

### 4.1 Limitations

The study was designed as an initial investigation into the benefits of a new silicone‐based patient‐side adhesive for pulse oximetry sensors. Gentleness of the adhesive was assessed on an elderly population with the intent to target fragile skin. However, no screening was performed on subjects to characterize fragile skin, and no age restrictions were used for the reapplication study. Further, pediatric patients were not included in the study population. The protein assay results for the silicone‐based adhesive were at the detection limit. Hence, the expected amount of protein removed by the silicone‐based adhesive would be lower than what was measured.

The 180° peel test was used to assess reapplication. This testing assumes that stiffness is the same throughout the device backing. Although the stiffness between the optical and tail regions did differ, as seen in the bimodal peel profile, the adhesive coverage was uniform across the respective devices. Further, the decay curves for the tail and optical regions demonstrated the same dynamics.

The sensor dwell time during the gentleness study was 4 h, timing that may be seen in the clinical setting. However, due to the time restraints of completing 18 reapplication trials, the dwell time of the sensors during this testing was only a few minutes. Clear differences between the two sensors were seen at six reapplications, demonstrating the superiority of the OxySoftN to MaxN in terms of reapplications.

## 5 CONCLUSION

Silicone adhesive can be a good solution for strong, yet gentle adhesives that are repositionable. The current study demonstrates that the OxySoftN sensor made of silicone had greater repositionability and less skin removal compared to the traditional MaxN sensor with acrylic and synthetic rubber adhesives. TEWL shows that there was not damage to the skin barrier function during removal of the device. Protein removal results show that they are more sensitive to changes in adhesives than TEWL and that OxySoftN removes a significantly less amount of skin compared to MaxN. Reapplication results show that the decay curves of the adhesives are markedly different and that the OxySoftN can withstand 18 repositions to skin, potentially more. These three metrics combined show that the new adhesive is gentler, as well as having the ability to stay adhered to the patient for a larger number of repositions.

## CONFLICT OF INTEREST

Jacob Dove and Derek Moody are employees of Medtronic. Other authors have no conflict of interest.

## Data Availability

The data that support the findings of this study are available from the corresponding author upon reasonable request.
